# Type of ANCA May Be Indispensable in Distinguishing Subphenotypes of Different Clinical Entities in ANCA-Associated Vasculitis

**DOI:** 10.3390/life12101467

**Published:** 2022-09-21

**Authors:** Afroditi Maria Konstantouli, Georgios Lioulios, Stamatia Stai, Eleni Moysidou, Asimina Fylaktou, Aikaterini Papagianni, Maria Stangou

**Affiliations:** 1 Department of Nephrology, Hippokration General Hospital, Aristotle University of Thessaloniki, 54642 Thessaloniki, Greece; 2 Department of Immunology, National Histocompatibility Center, Hippokration General Hospital, 54642 Thessaloniki, Greece

**Keywords:** ANCA-associated vasculitis, proteinase 3, myeloperoxidase, clinical phenotype, outcome

## Abstract

**Simple Summary:**

ANCA specificity seems to correlate more accurately with the symptomatology and disease outcome in ANCA-associated vasculitis in comparison to the classification based on clinical syndromes, as described in the Chapel Hill Consensus. This review analyzes the main differences in epidemiology, pathogenesis, histological and clinical manifestations and treatment response according to ANCA type.

**Abstract:**

The traditional nomenclature system for classifying antineutrophil cytoplasmic antibody (ANCA)-associated vasculitides (AAV) based on clinical phenotype describes granulomatosis with polyangiitis (GPA), eosinophilic granulomatosis with polyangiitis (EGPA) and microscopic polyangiitis (MPA) as distinct clinical entities. This classification has proved its expedience in clinical trials and everyday clinical practice; yet, a substantial overlap in clinical presentation still exists and often causes difficulties in prompt definition and clinical distinction. Additionally, new insights into the AAV pathogenesis point out that PR3 and MPO-AAV may not represent expressions of the same disease spectrum but rather two distinct disorders, as they display significant differences. Thus, it is supported that a classification based on ANCA serotype (PR3-ANCA, MPO-ANCA or ANCA-negative) could be more accurate and also closer to the nature of the disease compared to the phenotype-based one. This review aims to elucidate the major differences between PR3 and MPO-AAV in terms of epidemiology, pathogenesis, histological and clinical manifestations and response to therapeutic approaches.

## 1. Introduction

Antineutrophil cytoplasmic antibody (ANCA)-associated vasculitides (AAV) form a group of autoimmune, systemic, small-vessel vasculitides. Based on the 2012 Revised International Chapel Hill Consensus, the AAV group consists of four clinicopathologic entities: granulomatosis with polyangiitis—GPA (formerly known as Wegener’s granulomatosis), microscopic polyangiitis—MPA, eosinophilic granulomatosis with polyangiitis—EGPA (formerly known as Churg–Strauss syndrome) and renal-limited ANCA-associated vasculitis—RLV [1]. Two main types of ANCA autoantibodies, directed against neutrophil granule components, are detected in the circulation: PR3-ANCA, with specificity for proteinase 3 (PR3), and MPO-ANCA, with specificity for myeloperoxidase (MPO) [2]. Typically, small-sized vessels are involved; glomerular and alveolar capillaries are usually affected, resulting in glomerulonephritis and pulmonary hemorrhage, respectively, followed by skin involvement, manifested as purpuric lesions [3,4]. Other organs, such as the heart, central nervous system and gastrointestinal tract, are less frequently affected. Systemic and general symptoms, such as low-grade fever or malaise, may be the initial manifestations, while multiple tissues are usually involved during disease progression.

Several studies report an important overlap in clinical features between the most common clinical syndromes, GPA and MPA. Therefore, there is a growing assumption that a disease classification based on ANCA serology and not on clinical syndromes could probably differentiate patients much more accurately in terms of symptomatology, outcome and response to different therapeutic approaches. The present review aims to elucidate the major differences between PR3-ANCA and MPO-ANCA regarding epidemiology, pathogenesis, histology, clinical manifestations and treatment outcome and to compare findings with previous classifications based on clinical syndromes.

## 2. Epidemiology

### 2.1. Age and Gender

Several studies have validated that the male-to-female ratio is higher in PR3-AAV than in MPO-AAV. A large, multinational cohort showed that the male gender was more common among PR3-ANCA patients (61.5% compared to 43% among MPO-ANCA patients, *p* < 0.001) [5]. Likewise, a Japanese study reported that the majority of PR3-GPA patients were of male gender, while MPO-GPA is predominated by the female gender [6].

Regarding age, MPO-ANCA patients are usually older, with a median age estimated as 67 years. compared to 60 years., the median age of PR3-ANCA patients [5,6,7].

### 2.2. Geographic Variation

**Figure 1:**
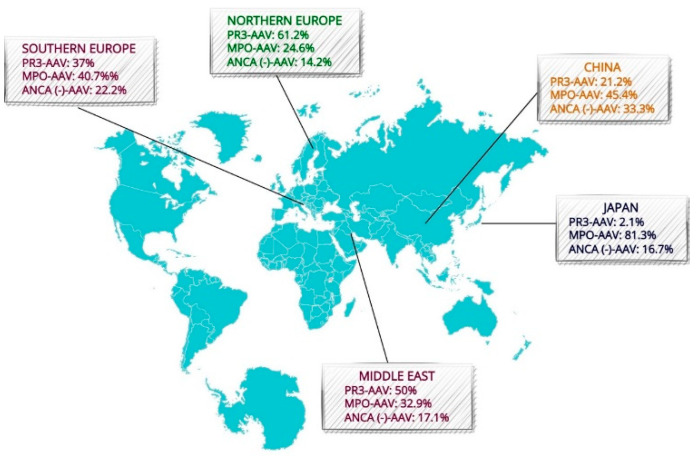
Geographic distribution of PR3-AAV, MPO-AAV and ANCA(−) AAV. Data obtained from Pearce et al. (‘Global, ethnic and geographic differences in the clinical presentations of anti-neutrophil cytoplasm antibody-associated vasculitis’). The Figure was partly generated using Servier Medical Art, provided by Servier, licensed under a Creative Commons Attribution 3.0 unported license (https://smart.servier.com (accessed on 1 August 2022)).

MPO and PR3-AAV differ significantly in geographic distribution [8] (Figure 1). MPO-ANCA specificity predominates in Asian populations; according to Pearce et al., in Japan, 81.3% of AAV patients were MPO-ANCA(+), whereas PR3-ANCA(+) accounted for only 2.1% of patients [9]. Similarly, a retrospective study of 467 AAV patients in China documented an MPO-ANCA predominance, with a frequency of 91.9% [10]. In contrast, PR3-ANCA positivity is more prevalent in Northern Europe and the Middle East and Turkey (61.2% and 50% of AVV, respectively) [9]. These geographic variations could reflect the effect of different ultraviolet (UV) radiation exposure [5] and distinct genetic backgrounds. The main allele associated with PR3-AAV, HLA-DPB1*04, has a higher frequency in European countries, compared to in Japan and China. Correspondingly, the HLA-DQ variants associated with MPO-AAV are more prevalent in East Asian populations [9].

## 3. Pathogenesis

### 3.1. Predisposing Factors

There is a body of growing evidence supporting that a genetically determined background, combined with exposure to environmental factors, can disrupt homeostasis and trigger ANCA production.

### 3.2. Genetic Associations

Genome-wide association studies have revealed that, although there is no evidence of inheritance in AAV, there is a clear genetic predisposition, associated more strongly with ANCA specificity than with the clinical syndromes [11]. Single-nucleotide polymorphisms (SNPs) in genes of the major histocompatibility complex (MHC) type II have been linked to AAV, with the HLA-DPB1*04 allele being strongly associated with the presence of ANCA [11,12]. PR3-AAV is associated with SNPs at the HLA-DP locus, especially with the HLA-DPB1*04 allele, and MPO-AAV with an SNP at the HLA-DQ locus. The presence of PR3-ANCA is also associated with an SNP at the gene encoding alpha-1-antitrypsin (SERPINA1) and variants of the gene encoding proteinase 3 (PRTN3) [11,12].

### 3.3. Environmental Factors

#### 3.3.1. Staphylococcus Aureus

It has been proposed that infections could induce loss of tolerance to self-antigens by molecular mimicry and excessive formation of neutrophil extracellular traps (NETs), which lead to prolonged exposure of autoantigens to immune cells [13,14,15]. Although chronic nasal carriage of St. aureus has been documented in MPO-ANCA vasculitis, it is mainly associated with PR3-ANCA patients, increasing their risk of relapse [16,17]. A clinical trial conducted by Salmela et al. found that chronic nasal carriage was almost exclusively a characteristic of PR3-ANCA vasculitis and, when detected in MPO-ANCA patients, was usually intermittent and not chronic [17]. Furthermore, the genetic make-up of St. aureus isolates seems to be different between PR3-ANCA and MPO-ANCA patients, indicating a possibility of distinct pathogenetic mechanisms [18].

**Silica:** Occupational silica exposure, related to mining, construction work, farming and agriculture, has been recognized as a risk factor for MPO-ANCA vasculitis [8,19]. In a population-based, case-control study, MPO-ANCA specificity was significantly more common than PR3-ANCA specificity among AAV patients with high exposure to silica (67% of MPO-ANCA patients were classified as having high exposure versus 40% of PR3-ANCA patients, *p* = 0.02) [20].

#### 3.3.2. Latitude and Ultraviolet (UV) Radiation

Epidemiological studies have proved an association between latitude and ANCA serotype. PR3-ANCA-positive vasculitis presents an inverse relation with vitamin-D-effective UV radiation levels. Its incidence increases while moving from south to north, as UV radiation levels decrease [5,21]. The protective role of UV radiation could be attributed to the immunomodulatory effects of vitamin D, which is believed to suppress Th1/Th17 cells and upregulate regulatory T cells [21].

#### 3.3.3. Drug-Induced Vasculitis

Medication is also associated with an ANCA-vasculitis-like syndrome. Drug-induced vasculitis has a milder phenotype, which recedes after discontinuation of the responsible drug [15,22]. MPO-ANCA specificity is more common, but concurrent MPO-ANCA and PR3-ANCA specificity can occur, predominantly in adults, after levamisole-adulterated cocaine inducement [23]. The list of implicated drugs is long and includes propylthiouracil (PTU) and other antithyroid drugs (carbimazole and methimazole), the anti-hypertensive agent hydralazine, the antibiotics minocycline and D-penicillamine and anti-TNFα agents [8,24].

### 3.4. Immune System Dysfunction

**Figure 2:**
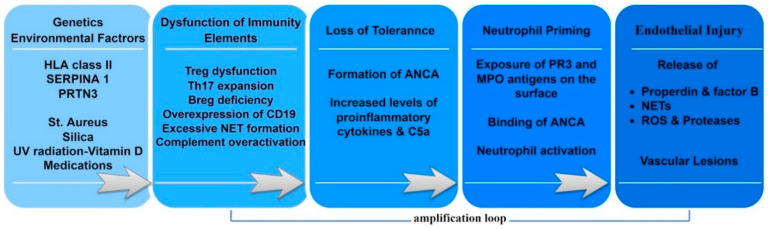
Pathogenetic events in ANCA-associated vasculitis. A genetically determined background, combined with exposure to environmental factors, can disrupt homeostasis and lead to dysfunction of the immune system. Without the suppressive supervision of Tregs, there is an abnormal expansion of Th17 and effector CD25+ T-cell populations. Furthermore, Breg deficiency leads to insufficient suppression of proinflammatory T-cell populations. Dysregulated expression of the CD19 coreceptor by a subset of memory B cells has also been reported. The impaired function of immunity results in elevated amounts of C5a and circulating cytokines, with the capacity to prime neutrophils. Primed neutrophils expose on their surface PR3 and MPO antigens. ANCAs bind to these antigens and activate neutrophils. Activated neutrophils release NETs and positive regulators of the alternative complement pathway (properdin and factor B), promoting complement hyperactivation and C5a formation. The released NETs, ROS and proteases damage the endothelium and provoke vascular lesions.

The precise steps triggering loss of self-tolerance to neutrophil antigens remain unknown. However, much attention has been drawn to the potential involvement of a variety of immune system components, including T and B lymphocytes, complement, neutrophil extracellular traps (NETs) and cytokine balance, as explicated below. Pathogenetic mechanisms and involvement of immune dysregulation are described in Figure 2.

#### 3.4.1. T Cells

Increasing evidence suggests an impaired Treg function [15,22,25]. Without the suppressive supervision of Tregs, there is an abnormal expansion of Th17 and effector CD25+ T-cell populations [25]. As a result, elevated levels of T-cell activity markers are detected in AAV patients, e.g., IL-2, IL-17 and IL-23 [22]. These interleukins perpetuate the release of further proinflammatory cytokines, such as TNF-α and IL-1b, that prime neutrophils for ANCA activation [15].

#### 3.4.2. B Cells

Dysregulation of B-cell function at various levels has been described. Wilde et al. showed that AAV patients have diminished levels of IL-10-producing B cells, which regulate T-cell activity (regulatory B cells, Breg) [26]. Breg deficiency leads to insufficient suppression of proinflammatory Th1 cells, promoting autoimmunity. Dysregulated expression of CD19 has also been documented [27]. A subset of memory B cells that express CD19 at higher levels shows loss of tolerance and autoreactivity.

#### 3.4.3. Complement

Stimulated neutrophils release properdin and factor B, positive regulators of the alternate complement pathway, resulting in C5a production [22]. C5a is a powerful chemoattractant that primes further neutrophils for ANCA-induced activation. Thus, the alternative complement pathway comprises an amplification loop [28]. In addition, plasma levels of factor H, a negative regulator of the alternative complement pathway (at the level of C3 convertase), are significantly lower in AAV patients, indicating complement hyperactivation [29].

A prospective study in AAV suggested that activation of the alternative complement pathway has an important role during disease development, but also influences progression [30]. Serum levels of C5a, C3a and Bb were increased and also had a significant correlation with the severity of histologic findings.

#### 3.4.4. NETs

Excessive NET formation, combined with impaired degradation, has a key role in pathogenesis [24,31,32]. NETs constitute an extracellular structure of fibers composed of granule proteins and chromatin. They are released from activated neutrophils and serve antimicrobial purposes by neutralizing a broad spectrum of pathogens [32]. MPO and PR3 antigens are found among the granular components of NETs, and their attachment to chromatin fibers possibly modifies their antigenicity [33]. Furthermore, defective degradation of NETs due to reduced activity of the enzyme DNase I prolongs the exposure of PR3 and MPO antigens and favors their detection by autoreactive, antigen-presenting cells [31,33,34]. ANCAs are thought to perpetuate NET formation by upregulating the production of IL-8, an important cytokine in neutrophil activation and NET generation [34].

#### 3.4.5. Cytokines

The impaired function of innate and adaptive immunity results in elevated amounts of inflammatory mediators, such as IL-6, IL-15, TGF-b1, VEGF, MCP-1 and MIP-1b. Measurement of these cytokines in the serum and urine of AAV patients demonstrated that some of them, such as EGF, IL-2 and IL-9, may have a favorable role, while others, e.g., IL-6, VEGF, IL-15, TGF-β1, MCP-1 and MIP-1β, have a detrimental effect on renal function [35]. Interestingly, there was a discrepancy between PR3 and MPO-AAV in cytokine production. MPO-ANCA patients had increased urinary levels of IL-17, while IL-6, BAFF and IL-5 predominated in the presence of PR3-ANCA.

Likewise, Berti et al. identified distinct profiles of serum circulating cytokines for PR3-AAV and MPO-AAV. PR3-AAV presented significantly higher levels of nine cytokines (IL-6, GM-CSF, IL-15, IL-18, CXCL8/IL-8, CCL-17/TARC, IL-18BP, sIL-2Ra, NGFb), whereas four cytokines were detected at higher levels in MPO-AAV (sIL6R, sTNFRII, NGAL, sICAM-1) [36].

## 4. Classification

**Figure 3:**
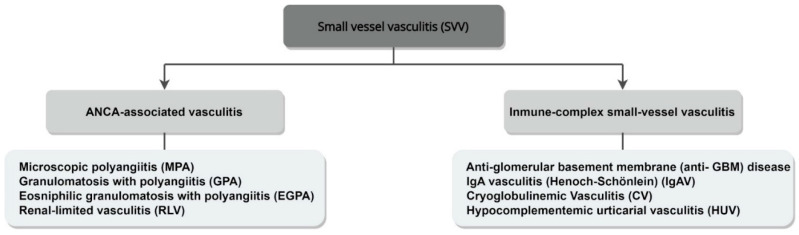
Classification of small-vessel vasculitis (SVV), based on immune deposits (CHCC 2012). SVV can be divided into ANCA-associated vasculitis, with few or no immune deposits, and immune complex SVV, with immunoglobin or complement deposits in vessel walls.

Classifying ANCA-associated vasculitides proves to be a real challenge due to their heterogeneity and significantly overlapping clinical features [37]. One of the most widely used systems is the 2012 Revised Chapel Hill Consensus Conference (CHCC 2012), which provides clear definitions for AAV and highlights the importance of ANCA serology [1]. This nomenclature system categorizes vasculitides based on the size of the affected blood vessels and the clinicopathological findings. AAVs are defined as necrotizing, small-vessel vasculitides (SVV), affecting small intraparenchymal arteries, arterioles, capillaries and venules. The absence of immune deposits (pauci-immune vasculitis) differentiates them from the immune complex SVV group (Figure 3). Furthermore, single-organ AAV (mainly renal-limited, RLV) is introduced as a fourth entity in the group of ANCA-associated vasculitides. The issue of ANCA(−) AAV is also addressed, with it being attributed to an ANCA-independent pathogenesis or to a group of ANCAs undetectable by the currently used methods. Recognizing the prognostic value of ANCAs, the use of a prefix to indicate ANCA serology is advocated, e.g., PR3-GPA, MPO-MPA. Table 1 summarizes the 2012 CHCC definitions for AAV.

For disease activity assessment, the use of the Birmingham Vasculitis Activity Score (BVAS) is advocated. The score is calculated based on signs and symptoms attributable to active vasculitis in nine distinct organ systems [38]. A cross-sectional study for the validation of the new BVAS version (BVAS v.3) indicated a strong correlation between the BVAS score and disease status in AAV patients [39]. The BVAS index has also been shown to have a prognostic value. Patients with a high BVAS score present more relapses and higher mortality [38,40]. Haris et al. documented that PR3-ANCA patients are more likely to have a higher BVAS score and, thus, present a poorer outcome [40].

## 5. ANCA Biology

The major antigenic targets of ANCA are myeloperoxidase (MPO) and proteinase 3 (PR3), components of cytoplasmic neutrophil granules [41]. MPO-ANCA pathogenicity has been demonstrated in animal models in which the passive transfer of anti-MPO IgG caused necrotizing glomerulonephritis [42]. On the contrary, it has been difficult to confirm the pathogenic role of PR3-ANCA using animal models. Furthermore, there is a discordance between clinical syndromes and ANCA serology. Although GPA is usually associated with PR3-ANCA and MPA with MPO-ANCA, none of the ANCAs is pathognomonic of a clinical syndrome, as MPO-GPA and PR3-MPA can also occur, yet less frequently [41].

Lysosomal membrane protein 2 (LAMP-2), a protein found in neutrophil lysosomes, has also been proposed as an antigenic target of ANCA. In fact, autoantibodies directed against LAMP-2 (anti-LAMP-2) are frequently detected in both MPO and PR3-ANCA-associated glomerulonephritis, as well as in ANCA(−) glomerulonephritis [43,44,45]. New data support the potential pathogenic role of anti-LAMP-2 in renal lesions. Firstly, a membrane glycoprotein of glomerular endothelial cells is structurally related to LAMP-2 and, thus, forms a target of anti-LAMP-2. Furthermore, a sequence homology of LAMP-2 with a bacterial fimbrial protein called FimH is found in many Gram-negative bacteria [46].

## 6. ANCA as a Prognostic and Follow-Up Biomarker

The utility of ANCA titres for monitoring patients and predicting relapses is still a subject of debate. Thompson et al. reported that patients demonstrating a decrease in PR3-ANCA titres, evaluated by direct ELISA at 4 months after treatment initiation, were more likely to achieve remission in a short period of time [47]. Likewise, Fussner et al. described that an increase in PR3-ANCA levels during complete remission was accompanied by a higher risk of severe relapse within a year, especially among patients with renal involvement or alveolar hemorrhage [48]. Regarding MPO-ANCA, patients with higher titres presented significantly lower renal survival rates at a two-year follow up and were more likely to need permanent dialysis [49]. Thus, monitoring ANCA titres along the course of immunotherapy could help clinicians distinguish patients who are not responding to therapy or suffer a poorer prognosis.

## 7. Renal Involvement—Histopathology

Renal involvement usually produces pauci-immune, necrotizing glomerulonephritis, with only a few or no immune deposits under immunofluorescence staining [50]. In 2010, an international group of renal pathologists organized the histopathologic findings into four classes of renal lesions based on the proportion between healthy and affected glomeruli [51]:

- Focal class, if >50% of glomeruli are healthy;
- Crescentic class, if >50% of glomeruli present crescents;
- Sclerotic class, if >50% of glomeruli are sclerotic;
- Mixed class, with no predominance of a lesion phenotype (less than 50% normal, less than 50% crescentic, less than 50% sclerotic glomeruli).

The four distinct glomerular phenotypes respond to the severity of renal damage and have a prognostic value [51,52,53]. The focal class has the best renal survival prognosis, followed by the crescentic and mixed group. On the contrary, the sclerotic phenotype is associated with poor renal outcome.

MPO and PR3-ANCA glomerulonephritis (GN) differ notably in their histopathological presentation. MPO-ANCA GN presents a smaller fraction of healthy glomeruli and more sclerotic glomerular lesions. Interstitial fibrosis and tubular atrophy are also more pronounced in MPO-AAV [54,55]. ANCA(−) vasculitis also shows a higher percentage of sclerotic lesions and interstitial fibrosis, similar to the damages found in MPO-ANCA vasculitis [56]. A recent study that evaluated the association between ANCA specificity and histopathology findings revealed that 64% of PR3-ANCA patients had the crescentic phenotype and only 3% the sclerotic one [57]. On the other hand, renal histology in MPO-ANCA and ANCA(−) patients was similar, with only 28% having the crescentic and 46% the sclerotic phenotype. Table 2 summarizes the main pathogenetic and clinical differences between PR3 and MPO-AAV.

## 8. Extrarenal Manifestations—ENT, Lung and Ocular Involvement

ENT involvement is usually manifested as chronic granulomatous rhinosinusitis with nasal crusting and epistaxis and less often with septal perforation and saddle nose [58]. Recurrent otitis media and hearing loss can also occur [59]. ENT involvement is strongly associated with PR3-ANCA positivity [60,61]. Similar symptoms can occur in MPO-ANCA patients, but less frequently. A retrospective analysis of three international, multicenter trials showed that ENT involvement, presenting as nasal obstruction and accompanied by granulomatous sinusitis, was definitely more frequent in PR3 compared to in MPO-ANCA patients and also had significant correlation with the degree of renal function impairment and severity of renal histology [60].

Lungs are frequently affected in both PR3 and MPO-AAV. Studies reported that more than 75% of AAV patients have clinical or radiological evidence of lung involvement [62,63]. Central airway disease, in the form of thickening and stenosis, and nodular lesions are the main patterns described in PR3-AAV, whereas interstitial fibrosis, bronchiectasis and alveolar hemorrhage are more prevalent in MPO-AAV [62].

Ophthalmologic disease, such as scleritis and episcleritis, is another common manifestation of AAV and occurs with higher frequency in PR3-AAV than in MPO-AAV. Among the AAV patients with ocular disease followed at the Mayo Clinic from 2003 to 2013, 64% were PR3-ANCA(+), 21% were MPO-ANCA(+) and 15% tested negative for ANCA [64]. The most common symptoms experienced were ocular injection, eye pain and visual acuity loss.

**Figure 4:**
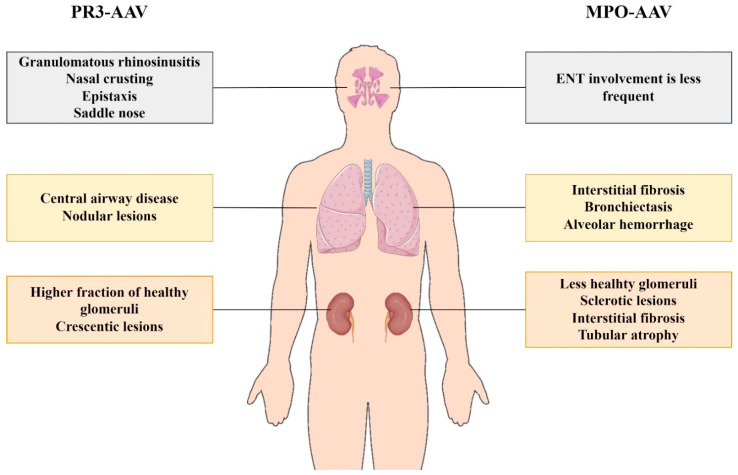
Characteristics of ENT, lung and kidney involvement in PR3-AAV and MPO-AAV. The Figure was partly generated using Servier Medical Art, provided by Servier, licensed under a Creative Commons Attribution 3.0 unported license (https://smart.servier.com (accessed on 1 August 2022)).

Characteristics of ENT, lung and kidney involvement are depicted in Figure 4.

## 9. Treatment

The treatment of AAV is based on immunosuppression and involves two phases: the induction phase, lasting between 3 and 6 months, and the maintenance phase once remission is achieved, switching to less toxic immunosuppressants for an additional period of 18–24 months [15,65].

Clinical trials in AAV and their results are summarized in Table 3.

### 9.1. Induction of Remission

For decades, oral cyclophosphamide combined with glucocorticoids was the first-line treatment for remission induction [66,67,68]. However, the severe, treatment-related toxicity, e.g., leukopenia and life-threatening opportunistic infections, hemorrhagic cystitis, malignancies and infertility, urged the development of safer protocols [69].

The CYCLOPS trial demonstrated that intravenous pulses of cyclophosphamide (*15 mg/kg every 2 to 3 weeks*) maintained the efficacy of its oral administration at a smaller cumulative dose, producing fewer episodes of severe leukopenia [70]. When long-term relapse data were collected retrospectively, intravenously treated patients had more relapses, but renal function and survival rates were similar in both regimen groups, offering promising results for pulse cyclophosphamide [71]. Interestingly, PR3-ANCA patients were at a higher risk of relapse when treated with cyclophosphamide pulses compared with MPO-ANCA patients, who responded equally to both regimens.

Two randomized trials concluded that rituximab is non-inferior to cyclophosphamide for remission induction. In the RAVE trial, rituximab (*375 mg/m^2^ of body surface per week for 4 weeks*) matched the efficacy of oral cyclophosphamide (*2 mg/kg per day for 3–6 months, followed by azathioprine*) in newly diagnosed disease, including severe renal involvement or alveolar hemorrhage, and was superior in relapsing disease [72]. The induction phase had a duration of 6 months. Regarding ANCA reactivity, PR3-ANCA patients treated with rituximab became ANCA(−) considerably more often than those receiving cyclophosphamide. A subsequent post hoc analysis of the data suggested that rituximab is superior to cyclophosphamide for remission induction in PR3-ANCA patients [73]. Both agents were associated with comparable rates of remission in MPO-ANCA patients.

The RITUXVAS trial compared rituximab with intravenous cyclophosphamide [74]. In this trial, the rituximab-based group also received two pulses of cyclophosphamide due to the relative lack of experience with rituximab at that time. As in the RAVE study, both immunosuppressants demonstrated similar efficacy in remission induction. A RITUXVAS follow-up study showed that mortality, relapses and end-stage renal disease did not differ significantly among patients with rituximab-induced remission without maintenance therapy (apart from with low-dose prednisolone) and cyclophosphamide-induced remission followed by azathioprine [75].

Despite the promising results regarding its efficacy, rituximab yielded similar rates of adverse effects with cyclophosphamide in both trials (RAVE and RITUXVAS), failing to provide any clear safety benefits.

### 9.2. Maintenance of Remission

A common practice for remission maintenance is converting to azathioprine after cyclophosphamide-induced remission.

The CYCAZAREM trial attempted to determine the optimal time interval for switching to azathioprine [76]. Patients were assigned to two groups, converting to azathioprine after 3–6 or 12 months of cyclophosphamide, respectively. Early cyclophosphamide replacement led to more relapses, but the difference was not found to be statistically important. Additionally, PR3-ANCA patients’ response to treatment did not differ significantly from the other group, although their frequency of relapse was higher than that of MPO-ANCA patients (61% versus 37% in the early replacement group and 41% versus 26% in the second group).

Another question concerned the optimal duration of remission maintenance therapy with azathioprine. The REMAIN study announced that prolonged azathioprine/prednisolone administration, beyond 24 months, increases both relapse-free survival and renal survival [77]. However, patients assigned to the prolonged therapy group were more likely to experience hematological and cardiovascular disorders compared to those who discontinued azathioprine at 24 months. On the contrary, Joode et al. reported that prolongation of azathioprine administration for more than 18 months did not significantly influence the relapse rates, and relapse-free survival depended more on the intensity of induction treatment and ANCA serology (e.g., more relapses in intravenous compared with oral cyclophosphamide and in PR3-ANCA compared with MPO-ANCA patients) [78].

Administration of rituximab for remission maintenance was also evaluated. The MAINRITSAN trial showed that rituximab infusions (*500 mg of rituximab on days 0 and 14 and at months 6, 12 and 18, plus daily prednisone*) were superior to oral azathioprine (*2 mg/kg/day for the first 12 months, followed by 1.5 mg/kg/day for 6 months and 1 mg/kg/day for 4 months, plus daily prednisone*) and reduced the frequency of severe relapses (5% in the rituximab group versus 29% in the azathioprine group) [79]. The rate of adverse effects was similar between the two groups.

Given that rituximab failed to reduce the treatment-related toxicity, the key question was whether a smaller cumulative dose remains effective whilst being less toxic. The MAINRITSAN 2 trial compared the standard infusions at fixed intervals (*at day 0, day 14 and at month 6, 12 and 18*) with tailored rituximab administration based on serological markers (monitoring of C19+ B-cell counts and ANCA levels every 3 months) [80]. Rituximab was reinfused only when one of these biomarkers reappeared or increased considerably. Evaluation at month 28 showed that relapse rates did not differ significantly between the personalized and the fixed-schedule regimen. Additionally, a cohort study conducted at the Mayo Clinic validated the efficacy of rituximab for remission induction and maintenance in PR3-ANCA patients with chronic, refractory disease and documented that reinfusing rituximab after a rise in B-cell count or ANCA titres could prevent an impending relapse [81].

### 9.3. Considerations before Treatment Selection

PR3-ANCA specificity, granulomatous ENT disease, St. aureus carriage and preserved renal function with low serum creatinine carry a higher relapse risk and might call for a more intense therapeutic strategy [15,67,82]. A higher cumulative dose during induction and prolongation of maintenance treatment are indicated [83].

## 10. Suggestions for Novel Classification of AAV

As previously defined clinical phenotypes seem not to be able to cover all aspects of AAV, there have been very important efforts to explore disease classification and describe novel subgroups. A remarkable approach was based on cluster analysis, performed in a large cohort of AAV patients. The present analysis started with GPA and MPA diseases and, after the clustering process, which involved multiple correspondence and hierarchical ascendant cluster analysis, resulted in classes with differences in the immune profile and clinical presentation, most significantly in patient outcome [84]. Even more recently, other investigators, again applying unbiased latent class analysis, identified a four-class AAV model, describing a distinct entity affecting young patients presenting with multiple organ involvement and followed by worse outcome [85]. All these attempts simply signify the huge spectrum of AAV, difficulties to define the specific entities and queries regarding treatment approach and outcome but may also indicate the presence of completely divergent diseases not distinctly identified yet.

For the present time, it seems that classification based on ANCA, including PR3-ANCA, MPO-ANCA and ANCA(−), is superior to clinical classification, e.g., GPA, MPA, EGPA and RLD, as it better correlates with disease pathogenesis, histology, clinical symptomatology and outcome.

## 11. Conclusions

Although the pathogenetic mechanisms of AAV are not yet fully understood, current evidence leads to the conclusion that PR3-ANCA and MPO-ANCA vasculitis differ significantly in prognosis and relapse risk. These differences reflect a need for a therapy tailored to each patient’s specific characteristics. Considering that PR3-ANCA serotype is associated with a higher relapse risk, these patients might need a higher cumulative dose to maintain remission, and, hence, oral cyclophosphamide might be more adequate than its intravenous administration. Furthermore, the data obtained through recent trials indicate that rituximab is a better option for remission induction and maintenance in PR3-ANCA patients. Regarding MPO-ANCA patients, azathioprine seems to be equal to rituximab for remission maintenance. The optimal duration of maintenance treatment remains a controversial issue and could be individualized based on serological biomarkers.

## Figures and Tables

**Table 1:** Definitions of the syndromic presentations of AAV, as established by the 2012 Revised International Chapel Hill Consensus Conference.

CHCC 2012 Name	CHCC 2012 Definition
Granulomatosis with polyangiitis (GPA)	Necrotizing granulomatous inflammation, usually involving the upper and lower respiratory tract, and necrotizing vasculitis, predominantly affecting small-to-medium vessels. Necrotizing glomerulonephritis is common.
Microscopic polyangiitis (MPA)	Necrotizing vasculitis, predominantly affecting small vessels. Necrotizing glomerulonephritis is very common. Pulmonary capillaritis often occurs. Granulomatous inflammation is absent.
Eosinophilic granulomatosis with polyangiitis (EGPA)	Eosinophil-rich and necrotizing granulomatous inflammation, often involving the respiratory tract, and necrotizing vasculitis, predominantly affecting small-to-medium vessels. Associated with asthma and eosinophilia.
Single-organ AAV	For example, renal-limited vasculitis (RLV). Vasculitis in arteries or veins of a single organ, without any features indicating that it is a limited expression of a systemic vasculitis.

**Table 2:** Differences between PR3-AAV and MPO-AAV in terms of epidemiology, pathogenesis, histology and clinical manifestations.

Feature	PR3-AAV	MPO-AAV
Male-to-female ratio	Higher	Lower
Age of onset	Younger	Older
Geographic distribution	Predominant in Northern Europe	Predominant in Japan and China
Genetics	SNPs at the HLA-DP locus HLA-DPB1*04 allele SERPINA 1 PRTN3	SNPs at the HLA-DQ locus
Environmental factors	St. Aureus Low exposure to UV radiation Vitamin D deficiency	Silica exposure
Cytokines	IL-6, GM-CSF, IL-15, IL-18, CXCL8/IL-8, CCL-17/TARC, IL-18BP, sIL-2Ra, NGFb, BAFF	sIL6R, sTNFRII, NGAL, sICAM-1
Ocular involvement	More common	Less common
ENT involvement	Very common	Less common
Pulmonary involvement	Central airway disease Nodular lesions	Interstitial fibrosis Bronchiectasis Alveolar hemorrhage
Renal histology	Higher fraction of healthy glomeruli Frequently focal and crescentic class	Smaller fraction of healthy glomeruli Frequently sclerotic class Interstitial fibrosis Tubular atrophy
Relapses	Higher risk	Lower risk
Renal survival	Better	Worse

Abbreviations: PR3, proteinase 3, MPO, myeloperoxidase, SNPs, single-nucleotide polymorphisms.

**Table 3:** Important clinical trials in the treatment of ANCA-associated vasculitis.

Clinical Trial	Objective	Results
CYCLOPS	Pulse CTX versus daily oral CTX for remission induction	Pulse CTX maintains the efficacy of oral CTX at a smaller cumulative dose
CYCLOPS long-term follow up	Long-term outcomes of the CYCLOPS study	MPO-ANCA patients respond equally to both regimes PR3-ANCA patients are at a higher risk of relapse when treated with CTX in pulses
RAVE	RTX versus oral CTX for remission induction	RTX matches the efficacy of oral CTX
RAVE post hoc analysis	Effect of ANCA specificity on treatment response	PR3-ANCA patients respond better to RTX than to oral CTX Comparable efficacy in MPO-ANCA patients
RITUXVAS	RTX versus CTX in pulses for remission induction	RTX matches the efficacy of CTX in pulses
Two-year follow up of the RITUXVAS study	Long-term outcomes of the RITUXVAS study	Outcome of death, ESRD and relapse did not differ significantly between the two regimen groups
CYCAZAREM	Optimal time interval to switch from CTX to AZT for remission maintenance	Early CTX replacement leads to more relapses, but the difference is not statistically important. PR3-ANCA patients relapse more often than MPO-ANCA patients after CTX suspension
REMAIN	Efficacy of two different durations of an AZT-based maintenance therapy	Prolonged maintenance therapy, beyond 24 months, reduces relapse risk and improves survival
IMPROVE	MMF versus AZT for remission maintenance	MMF is less effective than AZT for remission maintenance
MAINRITSAN	RTX versus AZT for remission maintenance	RTX is superior to AZT for maintenance of remission
MAINRITSAN2	Tailored, based on trimestral biological parameters versus fixed-schedule RTX infusions	Relapse rates were similar between the two administration regimens
MAINRITSAN2 post hoc analysis	Effect of omitting RTX administration at day 14	Relapse-free survival rates did not differ significantly
Rituximab for remission induction and maintenance in refractory GPA	Efficacy of RTX for maintenance of long-term remission in refractory GPA	RTX is effective for long-term maintenance of remission in PR3-ANCA patients. Monitoring B-cell levels can help to predict and prevent a relapse

Abbreviations: CTX, cyclophosphamide, AZT, azathioprine, RTX, rituximab, MMF, mycophenolate mofetil.
